# From the invasive front to organotropic pre-metastatic niches: spatial immune regulatory networks governing cholangiocarcinoma dissemination and metastasis-intercepting immunotherapy

**DOI:** 10.3389/fimmu.2026.1919864

**Published:** 2026-08-20

**Authors:** Longhao Zhang, Kai Zhang, Zhihong Chen, Xin Lu

**Affiliations:** Department of Liver Surgery, Peking Union Medical College Hospital, Chinese Academy of Medical Sciences and Peking Union Medical College, Beijing, China

**Keywords:** cancer-associated fibroblasts, cholangiocarcinoma, immunotherapy, invasive front, metastasis, pre-metastatic niche, spatial immunology, tumor microenvironment

## Abstract

Cholangiocarcinoma is an aggressive biliary tract malignancy in which metastatic relapse and primary or acquired resistance to immunotherapy remain major causes of mortality. Although immune checkpoint inhibitors have improved first-line treatment for advanced biliary tract cancer, most patients do not achieve durable benefit, indicating that immune failure is not explained by a single checkpoint pathway. In this Review, we propose a spatial immune-regulatory continuum for cholangiocarcinoma dissemination. Most direct single-cell and spatial evidence currently derives from intrahepatic cholangiocarcinoma, and its applicability to perihilar and distal disease remains to be established. This continuum begins in the tumor core and invasive front, where malignant cells, cancer-associated fibroblasts, tumor-associated macrophages, endothelial and lymphatic cells, regulatory T cells, immature neutrophils and excluded or dysfunctional cytotoxic T cells form a pro-invasive ecosystem. It then extends through extracellular vesicles, soluble mediators and lymphovascular routes that may educate organotropic pre-metastatic niches. Finally, lymph node, lung, liver, peritoneal and bone microenvironments provide organ-specific extracellular matrix, myeloid and stromal programs that enable immune evasion and metastatic colonization. By integrating clinical evidence, multi-omics studies, single-cell and spatial transcriptomics, extracellular vesicle biology, pre-metastatic niche concepts and emerging therapeutic strategies, we argue that cholangiocarcinoma metastasis should be targeted before overt dissemination whenever possible. In this Review, “metastasis-intercepting immunotherapy” is used as an author-defined conceptual framework for strategies intended to prevent or disrupt the immune–stromal conditions that enable dissemination and colonization, rather than merely shrink established metastatic lesions. Metastasis-intercepting immunotherapy will likely require rational combinations that reprogram the invasive front, restore dendritic-cell-mediated antigen presentation, block tumor-stroma-myeloid circuits, disrupt EV-mediated communication that may contribute to niche formation and select patients using spatial biomarkers rather than bulk immune markers alone.

## 1 Introduction: metastasis as an immune-spatial problem in cholangiocarcinoma

Cholangiocarcinoma (CCA) comprises intrahepatic, perihilar and distal tumors arising from the biliary epithelium and its related progenitor compartments. Its incidence and mortality vary substantially across regions, but a consistent clinical feature is late diagnosis, a high rate of recurrence after potentially curative surgery and frequent metastatic dissemination. Contemporary reviews and disease primers have emphasized that CCA is not a single disease: anatomical site, etiology, histologic growth pattern and molecular alterations all shape prognosis and treatment opportunities (1, 2). Large clinicopathologic and epidemiologic studies have also shown that CCA represents an increasing global burden, particularly for intrahepatic CCA, and that geographical heterogeneity reflects a mixture of inflammatory, infectious, metabolic and environmental risk factors (3, 4).

Therapeutic progress has been meaningful but incomplete. Cisplatin plus gemcitabine became the first-line systemic backbone after ABC-02, while FOLFOX offered a modest second-line survival advantage in ABC-06 (5, 6). Molecularly selected therapies, including FGFR inhibitors for FGFR2 fusions or rearrangements and IDH1 inhibition for IDH1-mutant disease, established the principle that a subset of advanced CCA is targetable (7, 8). More recently, durvalumab plus gemcitabine-cisplatin in TOPAZ-1 and pembrolizumab plus gemcitabine-cisplatin in KEYNOTE-966 moved immune checkpoint blockade into the first-line setting for advanced biliary tract cancer (9, 10).

Despite these advances, most patients with advanced CCA do not have long-lasting immune control. Median survival improvements in unselected populations remain modest, and response heterogeneity suggests that immunotherapy resistance is driven by the structure and function of the tumor microenvironment (TME), not only by tumor cell-intrinsic antigenicity. Existing reviews have summarized CCA immunology, immunotherapy trials and stromal biology in detail, but they usually organize the field by immune cell type or treatment class (11, 12). A complementary framework is needed for the specific problem of metastasis: where in the tumor does dissemination begin, how are distant organs conditioned, and which immune-regulatory circuits are most relevant for early interception?

This Review uses a spatial logic. We define the invasive front as a functional immune checkpoint, the pre-metastatic niche as a distant organ state induced before or during tumor cell arrival, and metastatic colonization as an organ-specific process of immune escape and stromal adaptation. This perspective aligns with the concept that metastasis is an inefficient, multi-step evolutionary and ecological process rather than a single late event (13, 14). It also fits the broader biology of pre-metastatic niche formation, in which tumor-secreted factors, extracellular vesicles (EVs), bone marrow-derived cells, vascular permeability and extracellular matrix (ECM) remodeling cooperate to prepare organ-specific soil for disseminated cancer cells (15, 16).

## 2 Rationale and scope of a spatial metastasis framework

Recent reviews have comprehensively summarized the immune-cell composition and heterogeneity of CCA, immune-checkpoint blockade, vaccine and cellular therapies, biomarker development and rational combination strategies (11, 12, 17, 18). Separate reviews have emphasized the desmoplastic tumor microenvironment, CAF biology, ECM remodeling and tumor–stroma crosstalk (19, 20). These studies have established the major cellular, stromal and therapeutic components of CCA immunobiology. However, the literature has generally been organized by cell type, signaling pathway or treatment class rather than by the sequential spatial stages through which dissemination and metastatic colonization occur. The present Review therefore examines how immune and stromal programs are reorganized from the tumor core and invasive front through lymphovascular dissemination to organ-specific metastatic niches, and how these transitions may create opportunities for metastasis interception.

This stage-based perspective is particularly relevant because the CCA TME is not spatially uniform: tumor core, peritumoral liver, biliary epithelium, portal tracts, invasive leading edge, lymphatic routes and distant organs represent distinct ecological compartments. Immune cells may be abundant in one compartment but excluded from another, and a bulk transcriptome cannot distinguish cytotoxic lymphocytes physically separated from malignant cells by CAF-rich ECM from lymphocytes positioned at productive tumor-immune interfaces. The immune contexture concept developed in other cancers shows that location, density and functional orientation of immune infiltrates can be as important as their absolute abundance (21, 22).

Spatial biology is particularly important in CCA because the disease arises in organs and ducts marked by chronic inflammation, cholestasis, fibrosis and specialized epithelial-mesenchymal interactions. Intrahepatic CCA often grows in a liver already exposed to injury, cholangitis, steatohepatitis, viral hepatitis or parasitic infection, while extrahepatic disease develops within a ductal and periductal stromal environment. The biliary tract is also exposed to bile acids, microbial products and mechanical obstruction, all of which can shape immune and stromal responses. These features suggest that metastasis in CCA is not simply tumor-cell dissemination, but an interaction between malignant programs and pre-existing or tumor-induced spatial tissue states (23, 24).

**Figure 1:**
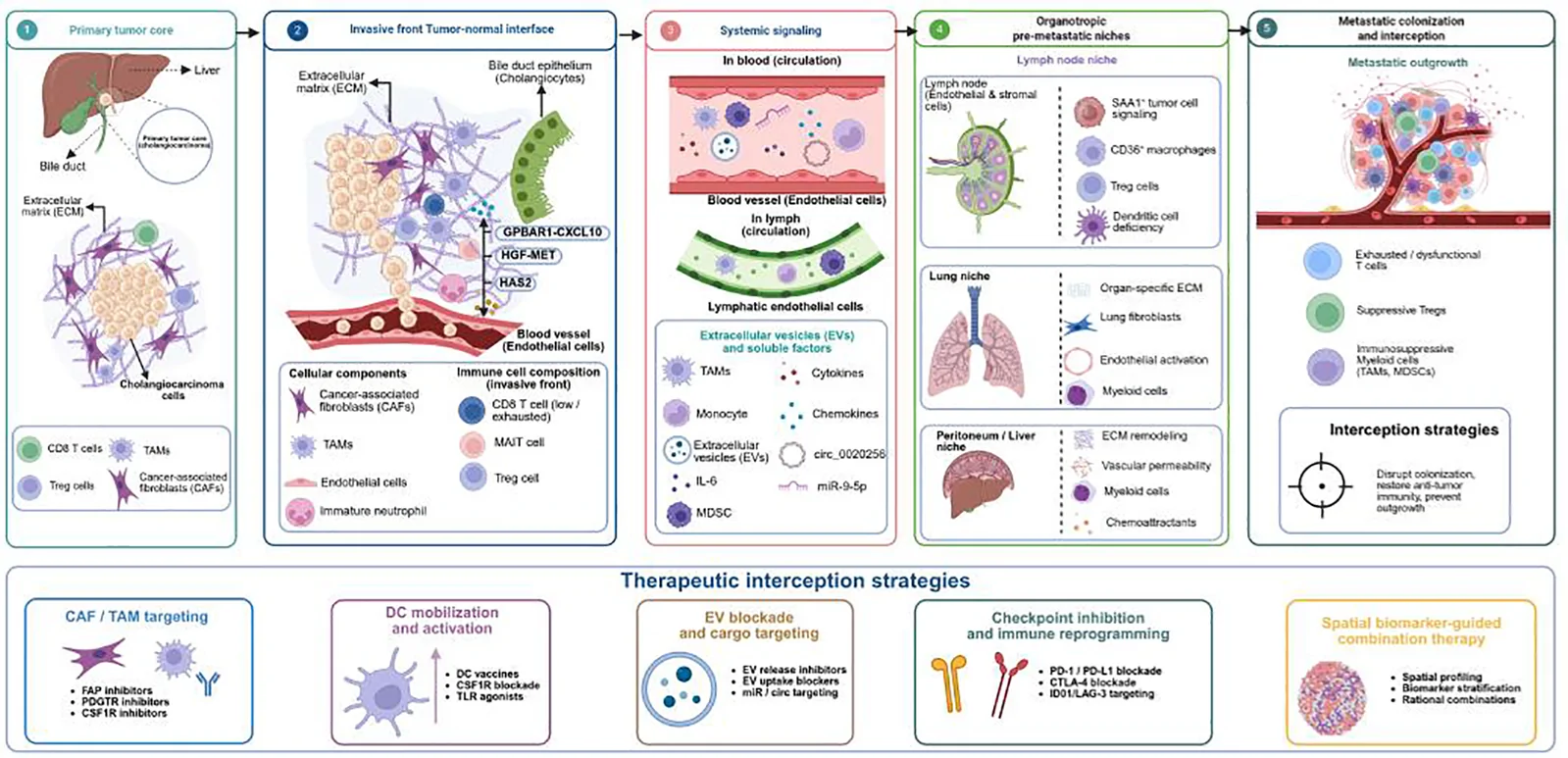
Conceptual spatial immune-regulatory continuum of cholangiocarcinoma dissemination. This schematic summarizes cholangiocarcinoma dissemination as a spatially ordered immune-regulatory continuum. Direct single-cell and spatial support for several components of this continuum derives predominantly from iCCA, whereas their applicability to perihilar and distal CCA remains to be established. In the primary tumor core, cholangiocarcinoma cells are embedded in a desmoplastic extracellular matrix enriched with cancer-associated fibroblasts, tumor-associated macrophages, regulatory T cells and variably positioned CD8 T cells. At the invasive front, tumor cells interact with CAFs, TAMs, endothelial cells, immature neutrophils, dendritic cells and lymphatic structures, generating a tumor–normal interface characterized by stromal remodeling and immune exclusion, with candidate programs including GPBAR1–CXCL10 signaling alongside HGF–MET signaling and HAS2-associated matrix deposition. Tumor-derived extracellular vesicles, cytokines and chemokines are depicted as entering blood and lymphatic routes and may contribute to systemic myeloid education and the recruitment of monocytes, TAMs and MDSCs. These circulating signals may contribute to putative organotropic niches in lymph nodes, lung and peritoneal or liver sites; however, direct evidence that these organs are conditioned before CCA-cell arrival is currently lacking. Established metastatic lesions evolve into cold immune ecosystems dominated by exhausted or dysfunctional T cells, suppressive Tregs and immunosuppressive myeloid cells. The lower panels highlight candidate metastasis-interception strategies, including CAF/TAM targeting, dendritic-cell mobilization and activation, EV blockade or cargo targeting, checkpoint inhibition, immune reprogramming and spatial biomarker-guided combination therapy. Among these approaches, checkpoint inhibitor–chemotherapy combinations have clinical support in advanced biliary tract cancer, whereas the niche-directed applications depicted here remain investigational. CAF, cancer-associated fibroblast; CCA, cholangiocarcinoma; circ, circular RNA; CSF1R, colony-stimulating factor 1 receptor; CTLA-4, cytotoxic T-lymphocyte-associated protein 4; CXCL10, C-X-C motif chemokine ligand 10; DC, dendritic cell; ECM, extracellular matrix; EV, extracellular vesicle; FAP, fibroblast activation protein; GPBAR1, G protein-coupled bile acid receptor 1; HAS2, hyaluronan synthase 2; HGF, hepatocyte growth factor; IDO1, indoleamine 2,3-dioxygenase 1; LAG-3, lymphocyte activation gene 3; MAIT cell, mucosal-associated invariant T cell; MDSC, myeloid-derived suppressor cell; MET, MET receptor tyrosine kinase/HGF receptor; miR, microRNA; PD-1, programmed cell death protein 1; PD-L1, programmed death-ligand 1; PDGFR, platelet-derived growth factor receptor; TAM, tumor-associated macrophage; TLR, Toll-like receptor; Treg, regulatory T cell.

The central thesis of this Review is that CCA metastasis is governed by a spatial immune-regulatory network connecting four levels: the desmoplastic tumor core, the invasive front, lymphovascular dissemination and organotropic niches. Each level has distinct but interacting cellular modules, including CAFs, tumor-associated macrophages (TAMs), dendritic cells (DCs), regulatory T cells (Tregs), neutrophils, myeloid-derived suppressor cells (MDSCs), endothelial cells, lymphatic endothelial cells (LECs) and malignant subclones. The practical value of this model is therapeutic. In this Review, we use the term “metastasis-intercepting immunotherapy” as an author-defined conceptual framework for strategies intended to prevent or disrupt the immune–stromal conditions that enable dissemination and colonization, rather than merely shrink established metastatic lesions. This framework highlights intervention windows before macroscopic metastases become established. The overall framework is summarized in **Figure 1**.

### 2.1 Literature search strategy and evidence prioritization

This narrative Review was informed by searches of PubMed, Web of Science and Google Scholar from database inception through June 30, 2026, with the search restricted to English-language publications. Search concepts included “cholangiocarcinoma,” “intrahepatic cholangiocarcinoma,” “perihilar cholangiocarcinoma” and “distal cholangiocarcinoma,” combined with terms related to the “tumor microenvironment,” “spatial transcriptomics,” “single-cell RNA sequencing,” “invasive front,” “metastasis,” “pre-metastatic niche,” “extracellular vesicles,” “cancer-associated fibroblasts,” “tumor-associated macrophages,” “neutrophils,” “dendritic cells” and “immunotherapy.” Reference lists of relevant primary studies and authoritative reviews were also screened manually. Because this was a narrative rather than a systematic review, no formal meta-analysis or risk-of-bias scoring was undertaken. Evidence was prioritized hierarchically: first, human CCA studies incorporating single-cell, spatial, paired primary-metastatic or clinically annotated data; second, CCA-specific mechanistic studies using patient-derived materials or experimental models; and third, evidence from other cancer types, which was used only to identify transferable biological principles or to generate testable hypotheses. Findings restricted to intrahepatic CCA were labeled as iCCA-specific, whereas mechanisms supported by a single study or extrapolated from other malignancies were described as preliminary, proposed or inferred rather than established.

## 3 Clinical heterogeneity and the metastatic phenotype of CCA

CCA heterogeneity begins with anatomy. Intrahepatic CCA, perihilar CCA and distal CCA differ in risk factors, molecular alterations, patterns of local spread and surgical options. Molecular profiling has identified therapeutically relevant alterations such as FGFR2 fusions, IDH1 mutations, ERBB2 amplification, BRAF mutations and DNA repair defects, but these alterations are unevenly distributed across anatomical subtypes (25, 26). Integrative genomic studies have also shown that etiologic context matters: fluke-associated, viral-associated and inflammation-associated tumors can carry distinct mutational and epigenomic landscapes (27, 28).

Metastatic behavior reflects this heterogeneity. Intrahepatic CCA may spread intrahepatically, invade vasculature, metastasize to regional lymph nodes and seed distant sites such as lung, peritoneum and bone. Perihilar and distal tumors commonly spread along lymphatic and perineural routes and can be difficult to control locally even without widespread hematogenous disease. Surgical series consistently show that lymph node metastasis, vascular invasion, multifocality and positive margins predict worse outcomes (29, 30). These clinical observations imply that metastasis-promoting niches are active early, including at the tumor-liver interface and in draining lymph nodes.

Molecular classification has provided additional layers of stratification. Early transcriptomic studies identified proliferation and inflammation classes in intrahepatic CCA, with the proliferation class enriched for receptor tyrosine kinase signaling and poorer prognosis (31, 32). Subsequent genomic and epigenomic analyses revealed actionable oncogenic dependencies as well as subgroups associated with immune and stromal pathways (33, 34). These classifications are useful, but they remain insufficient for predicting immune therapy response or metastatic trajectory because they often rely on bulk tumor samples that average across tumor core, stroma and immune compartments.

An important implication for this Review is that metastatic CCA should not be approached as a uniform endpoint. Lymph node spread, intrahepatic satellite formation, lung colonization, peritoneal dissemination and bone metastasis likely depend on partly different immune and stromal selection pressures. A spatial framework can therefore complement genomic precision oncology: oncogenic mutations may initiate or sustain tumor programs, while the invasive front and target-organ microenvironment determine which malignant cells can leave, survive and colonize. **Table 1** provides a stage-based evidence map that distinguishes established clinical observations and direct CCA/iCCA findings from candidate, single-study or cross-cancer inferences.

**Table 1:** Evidence map linking spatial immune biology to CCA dissemination.

Spatial stage/evidence domain	Key observation	Dominant circuit or cell state	Metastasis-focused interpretation	Selected refs
Clinical heterogeneity	CCA incidence, anatomy and etiology differ by intrahepatic, perihilar and distal subtype.	Anatomical site, inflammatory background and actionable genomic alterations	Metastasis models should be stratified by subtype rather than pooling all CCA.	(1–4)
Systemic treatment benchmark	Gemcitabine-cisplatin, second-line FOLFOX and selected targeted agents define current systemic baselines.	Chemotherapy backbone; FGFR2 and IDH1 vulnerabilities	Metastasis-intercepting combinations must improve on contemporary systemic care.	(5–8)
Checkpoint era	Durvalumab or pembrolizumab with gemcitabine-cisplatin improves first-line outcomes but benefits remain heterogeneous.	PD-1/PD-L1 axis plus chemotherapy-induced immune modulation	Spatial biomarkers may explain which CCA ecosystems respond to ICI-containing regimens.	(9, 10, 35)
Bulk immune subtypes	iCCA contains immune-desert and inflammatory TME subtypes.	T-cell infiltration, checkpoint expression and stromal/immune state differences	Bulk immune classification provides a starting point for iCCA but cannot localize immune cells along the metastatic cascade.	(36, 37)
Single-cell tumor-stroma crosstalk	A single human iCCA scRNA-seq study reported malignant epithelial heterogeneity and a candidate tumor EV miR-9-5p–IL-6 communication axis involving vascular CAFs.	Candidate tumor EV–vascular CAF communication; IL-6 signaling	Single-study mechanistic evidence requiring independent validation.	(38, 39)
Molecular immune subtypes	S100P and SPP1 mark distinct iCCA subtypes with different immune infiltration patterns.	Macrophage, NK-cell and PD-1-positive CD8 T-cell differences	Lineage state and immune state should be integrated when evaluating metastatic potential.	(40, 41)
Treg and CD8 T-cell state	Multimodal iCCA profiling identifies insufficient CD39-positive tumor-reactive CD8 T cells and hyperactivated MEOX1-associated Tregs.	MEOX1-associated Treg program; limited tumor-reactive CD8 compartment	Local immune failure may reflect T-cell quality and position, not only T-cell abundance.	(42, 43)
Spatial macrophage crosstalk	One iCCA spatial transcriptomic study reported a candidate TFF3-associated tumor–macrophage interaction.	Candidate TFF3-related tumor–macrophage crosstalk; TAM polarization	Single-study spatial association requiring independent functional validation.	(44, 45)
Invasive-front microstructure	Leading-edge scRNA-seq and spatial transcriptomics identify proliferative tumor cells, POSTN+FAP+ fibroblasts, SPP1+ macrophages and low-function CD8 T cells in iCCA.	CAF–TAM–T-cell spatial neighborhood	Candidate immune-regulatory checkpoint at the invasive front.	(46–48)
CAF heterogeneity	Lineage tracing and scRNA-seq in iCCA models define CAF subpopulations associated with HAS2- and HGF–MET-related tumor-supportive programs.	myCAF-HAS2; iCAF-HGF/MET; HSC-derived CAFs	Specific CAF states, not CAFs as a bulk population, may organize metastatic tracks.	(49, 50)
Promigratory fibroblasts	LGALS1-positive fibroblasts promote iCCA proliferation and migration.	LGALS1; CCR2, ADAM15 and beta-integrin-related tumor programs	Fibroblast-targeted strategies should focus on functional states linked to invasion.	(51–53)
Macrophage education	Individual CCA studies have reported tumor-initiating macrophage education by stem-like cells and a candidate TAM-derived exosomal circ_0020256 axis.	TAM education; candidate circ_0020256/miR-432-5p/E2F3 axis	Proof-of-concept myeloid mechanisms requiring independent validation.	(45, 54, 55)
Lymphatic spread	Lymphangiogenesis and lymphatic vessel density associate with nodal metastasis and prognosis in biliary tumors.	VEGF-C/D-related lymphangiogenesis; lymphatic endothelial tolerance	Regional nodes are active immune organs, not merely staging stations.	(56–58)
DC-dependent nodal control	DC mobilization and activation reduce lymph-node metastasis in iCCA models.	Beta-catenin–CXCL12–DC axis	Direct preclinical rationale for restoring nodal antigen presentation.	(59–62)
EV and PMN biology	CCA EV studies show tumor-CAF and TAM-tumor communication; canonical PMN literature shows EVs can condition target organs.	EV cargo, exosome integrins, VEGFR1+ progenitors, endothelial leak and myeloid recruitment	Organ-specific PMN formation in CCA is plausible but still requires direct paired-organ proof.	(15, 16, 63–71)
Organ-specific ECM	Patient-derived CCA organoids respond differently to decellularized lung and lymph node ECM.	Target-organ ECM composition and stiffness	The metastatic niche can select epithelial state and should be modeled experimentally.	(72, 73)

Direct single-cell or spatial support for several programs—including POSTN+FAP+ CAFs, LGALS1+ fibroblasts, MEOX1-associated Tregs and DC deficits—derives predominantly from iCCA; their relevance to perihilar and distal CCA remains to be established. Abbreviations: CAF, cancer-associated fibroblast; CCA, cholangiocarcinoma; DC, dendritic cell; ECM, extracellular matrix; EV, extracellular vesicle; ICI, immune checkpoint inhibitor; iCCA, intrahepatic cholangiocarcinoma; PMN, pre-metastatic niche; TAM, tumor-associated macrophage; TME, tumor microenvironment.

## 4 Immune landscapes of CCA: from bulk subtypes to single-cell and spatial atlases

Bulk immune classification has established that iCCA includes both immune-cold and immune-inflamed states. A landmark iCCA study identified four TME-based immune subtypes, including an immune-desert group representing a substantial proportion of tumors and an inflammatory subtype enriched for T-cell infiltration, immune-checkpoint expression and better survival (36). This heterogeneity challenges the view that iCCA is uniformly non-immunogenic and may help explain the limited efficacy of unselected immune-checkpoint blockade. Comparable immune-subtype distributions in perihilar and distal CCA remain less well defined (37). Most single-cell and spatial studies discussed below likewise focus on iCCA.

Single-cell transcriptomics then began to resolve which cells create these states. One human iCCA single-cell study reported malignant epithelial heterogeneity and extensive intercellular crosstalk, including a candidate tumor EV miR-9-5p–IL-6 communication axis involving vascular CAFs (38). Another single-cell study proposed two molecularly distinct intrahepatic CCA subtypes marked by S100P and SPP1 expression, with different immune infiltration patterns involving macrophages, NK cells and PD-1-positive CD8 T cells (40). These studies indicate that immune phenotype is linked to malignant lineage programs and stromal architecture.

T cell states are also heterogeneous. Multimodal single-cell profiling of intrahepatic CCA found insufficient infiltration of CD39-positive tumor-reactive CD8 T cells and hyperactivated Tregs, including a MEOX1-associated Treg program linked to poor outcome (42). A separate immune-cell atlas of cholangiocarcinoma described functionally diverse lymphoid and myeloid compartments and highlighted the importance of cell-cell communication in shaping the tumor immune ecosystem (74). Together, these data suggest that improving CCA immunotherapy requires more than increasing T cell numbers; it requires restoring productive spatial interactions among antigen-presenting cells, tumor-reactive T cells and suppressive stromal compartments.

Spatial transcriptomics and spatially resolved pathology have begun to localize these cellular states. In a single iCCA spatial transcriptomic study, a candidate TFF3-associated tumor–macrophage interaction was reported, whereas a leading-edge single-cell and spatial atlas localized proliferative tumor cells, endothelial interactions, POSTN-positive FAP-positive fibroblasts and low-cytotoxicity CD8 T cells to the invasive margin (44, 46). Single-cell analysis of iCCA lymph-node metastases further identified a candidate interaction between SAA1-positive tumor cells and CD36-positive macrophages, while a recent single-cell multi-omics study reported metastasis-associated epithelial states (75, 76). Together, these early atlas-level studies indicate that spatially distinct epithelial, stromal and immune states emerge during iCCA progression, although their functional contributions to dissemination and metastatic colonization remain to be established.

## 5 The invasive front as a spatial immune checkpoint

The invasive front is the interface where malignant glands, surrounding liver or biliary tissue, CAF-rich ECM, blood and lymphatic vessels, nerve bundles and infiltrating immune cells physically meet. In many epithelial cancers, invasive-front immune architecture is prognostically informative because it captures tumor-immune confrontation more directly than the tumor core. This principle has been formalized in colorectal cancer immune scoring and in broader studies of immune contexture (47, 48). In CCA, the invasive front is especially relevant because the tumor often grows within a fibrotic liver or periductal stroma that can both restrain and facilitate invasion depending on its composition.

**Figure 2:**
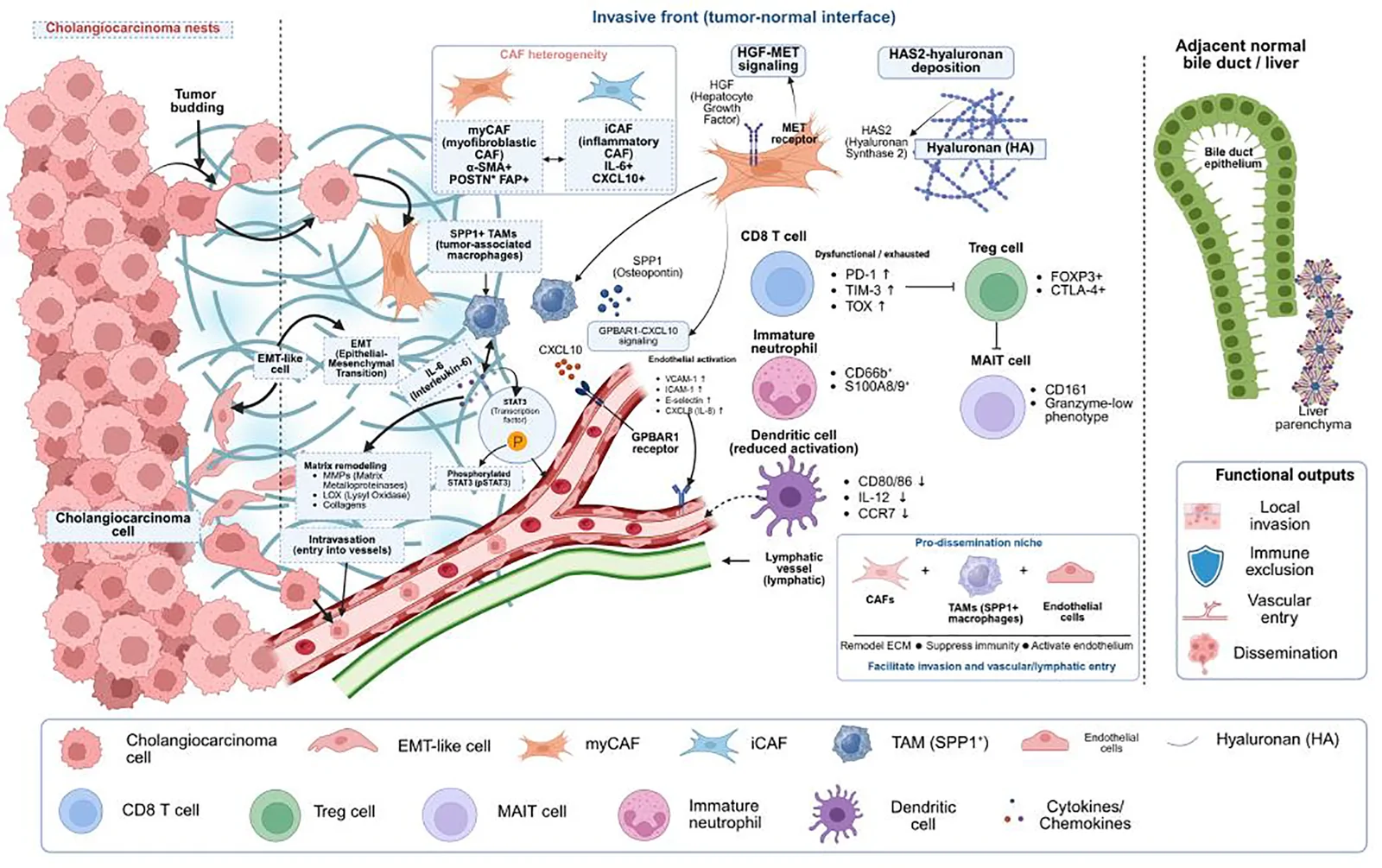
The invasive front as a spatial immune checkpoint in cholangiocarcinoma. This schematic integrates iCCA leading-edge and CAF data with broader CCA and cross-cancer evidence. Direct iCCA evidence supports the spatial enrichment of POSTN-positive FAP-positive fibroblasts, SPP1-positive macrophages and dysfunctional CD8 T cells at the leading edge, as well as distinct CAF states. The specific POSTN-positive FAP-positive CAF–SPP1-positive TAM–MAIT cell triad and the bile acid–GPBAR1–CXCL10 axis each derive from a single recent study; their integration with IL-6/STAT3 signaling, endothelial remodeling and immune exclusion into a causal dissemination network remains hypothesis-generating and requires independent functional validation. The invasive front represents a functional tumor–normal interface where cholangiocarcinoma nests, adjacent bile duct or liver tissue, stromal cells, immune cells and vascular structures converge. Tumor budding and EMT-like tumor cells emerge from cholangiocarcinoma nests and interact with heterogeneous CAF populations, including myCAF-like and iCAF-like states. These fibroblast states produce matrix-remodeling programs involving collagens, MMPs, LOX and hyaluronan, while HGF–MET and HAS2–hyaluronan signaling provide trophic and structural support for invasion. SPP1-positive TAMs and IL-6/STAT3 signaling are depicted as potentially reinforcing tumor–stroma crosstalk, endothelial activation and immune suppression. In the proposed model, candidate GPBAR1–CXCL10 signaling may link the biliary microenvironment to endothelial and myeloid remodeling and may promote immature neutrophil recruitment and a pro-dissemination niche. At the same interface, dysfunctional or exhausted CD8 T cells, Tregs, MAIT cells, immature neutrophils and poorly activated dendritic cells are depicted as a candidate immunosuppressive barrier that may limit effective antitumor immunity. This proposed invasive-front ecosystem may facilitate local invasion, immune exclusion, vascular or lymphatic entry and subsequent dissemination. α-SMA, alpha-smooth muscle actin; CAF, cancer-associated fibroblast; CD8, cluster of differentiation 8; CD66b, cluster of differentiation 66b; CD80/86, cluster of differentiation 80/86; CD161, cluster of differentiation 161; CCA, cholangiocarcinoma; CCR7, C-C motif chemokine receptor 7; CTLA-4, cytotoxic T-lymphocyte-associated protein 4; CXCL8, C-X-C motif chemokine ligand 8; CXCL10, C-X-C motif chemokine ligand 10; DC, dendritic cell; ECM, extracellular matrix; EMT, epithelial-mesenchymal transition; FAP, fibroblast activation protein; FOXP3, forkhead box P3; GPBAR1, G protein-coupled bile acid receptor 1; HA, hyaluronan; HAS2, hyaluronan synthase 2; HGF, hepatocyte growth factor; ICAM-1, intercellular adhesion molecule 1; iCAF, inflammatory cancer-associated fibroblast; IL, interleukin; LOX, lysyl oxidase; MAIT cell, mucosal-associated invariant T cell; MET, MET receptor tyrosine kinase/HGF receptor; MMP, matrix metalloproteinase; myCAF, myofibroblastic cancer-associated fibroblast; PD-1, programmed cell death protein 1; POSTN, periostin; pSTAT3, phosphorylated signal transducer and activator of transcription 3; S100A8/9, S100 calcium-binding proteins A8 and A9; SPP1, secreted phosphoprotein 1; STAT3, signal transducer and activator of transcription 3; TAM, tumor-associated macrophage; TIM-3, T-cell immunoglobulin and mucin-domain containing-3; TOX, thymocyte selection-associated high mobility group box; Treg, regulatory T cell; VCAM-1, vascular cell adhesion molecule 1.

The leading-edge single-cell and spatial transcriptomic study mentioned above provides direct evidence that the iCCA invasive front is not simply a histologic border. It is enriched for proliferative tumor cells, endothelial interactions and POSTN-positive FAP-positive CAFs, while cytotoxic T cells show reduced effector function and exhaustion-like states (46). That single recent iCCA study proposed a local triad involving mucosal-associated invariant T (MAIT) cells, SPP1-positive macrophages and POSTN-positive CAFs that may promote tumor progression. Because this triad has not been independently validated, **Figure 2** presents it as a hypothesis-generating spatial model rather than an established CCA mechanism.

CAFs are central to this checkpoint. Intrahepatic CCA is one of the most desmoplastic liver tumors, and CAFs can originate from hepatic stellate cells, portal fibroblasts, bone marrow-derived precursors and other mesenchymal sources. In a Cancer Cell study using lineage tracing, single-cell sequencing and functional experiments, hepatic stellate cell-derived CAFs were identified as major CAF contributors in intrahepatic CCA, with myCAF and iCAF subpopulations promoting tumor growth through HAS2 and HGF-MET-related programs (49). Another single-cell study defined LGALS1-positive fibroblasts that can promote intrahepatic CCA proliferation and migration through tumor-cell programs involving CCR2, ADAM15 and beta-integrin signaling (51).

Earlier mechanistic work already pointed to tumor-CAF reciprocity. CCA-derived platelet-derived growth factor-D can recruit and activate CAFs, while myofibroblast-derived platelet-derived growth factor-BB can promote Hedgehog survival signaling in CCA cells (24, 77). Hepatic myofibroblasts can also promote human CCA progression through epidermal growth factor receptor activation (78, 79). These studies indicate that invasion is sustained by reciprocal paracrine loops rather than by malignant cells alone.

The ECM produced by CAFs contributes to physical invasion and immune exclusion. Collagen deposition, lysyl oxidase-mediated crosslinking, hyaluronan accumulation and altered matrix stiffness can influence integrin signaling, epithelial-mesenchymal plasticity, vascular invasion and T cell migration (80, 81). In general cancer biology, ECM remodeling is known to regulate growth factor availability, mechanotransduction, angiogenesis and immune-cell trafficking (52, 53). In CCA, the intense desmoplastic response makes these ECM mechanisms particularly relevant, but future work must distinguish metastasis-promoting CAF states from CAF states that restrain tumor growth or maintain tissue architecture.

A single recent CCA preclinical study reported a candidate bile acid–CAF–neutrophil circuit in which high bile acid levels activated GPBAR1 on CAFs, induced CXCL10 secretion, enhanced EMT and metastasis, recruited neutrophils and maintained immature immunosuppressive neutrophil phenotypes; inhibition of this circuit improved anti-PD-1 activity in the reported models (82). This candidate circuit links anatomical exposure, CAF activation, neutrophil biology and immunotherapy resistance, but requires independent validation before it can be considered a general CCA mechanism. It also illustrates how the biliary context may generate immune–stromal interactions that differ from those in other gastrointestinal malignancies despite shared metastatic principles. Together, these findings identify the invasive front as a multicellular niche in which CAF state, ECM organization, myeloid recruitment and T-cell dysfunction converge.

## 6 Myeloid and lymphoid circuits that enable local escape

TAMs are consistently implicated in CCA progression. CCA stem-like cells can educate macrophages and shape a tumor-initiating niche, while TAM abundance and polarization have been associated with tumor growth, invasion and poor prognosis (45, 54). In mouse models, targeting TAMs and granulocytic MDSCs augments PD-1 blockade, supporting the concept that myeloid cells help define resistance to immune checkpoint inhibition (83, 84). Spatial studies now refine this view by suggesting that TAMs at specific tumor regions, not only total macrophage abundance, may be critical for invasion and metastasis.

SPP1-positive macrophages provide an example of regionally restricted myeloid states. The leading-edge iCCA atlas identified these cells within a proposed POSTN-positive CAF-rich neighborhood, whereas studies in liver and colorectal cancers have linked analogous SPP1-positive TAM programs to fibrosis, angiogenesis and immune suppression (46, 85, 86). These observations support, but do not yet establish, a role for this macrophage state in ECM remodeling, T-cell exclusion and lymphovascular entry in CCA.

Neutrophils and granulocytic MDSCs are another local escape module. In intrahepatic CCA, CXCL5 can recruit intratumoral neutrophils and contribute to metastasis and recurrence, while IL-17-positive cells and neutrophils have been associated with prognosis (87, 88). Broader cancer literature shows that neutrophils can promote metastasis by secreting proteases, reactive oxygen species, angiogenic factors and neutrophil extracellular traps (NETs), and by suppressing T cells (89, 90). In CCA, the candidate bile acid–GPBAR1–CXCL10 pathway reported in a single recent preclinical study provides a disease-contextualized example of how stromal signaling may sustain a neutrophil-dominant immunosuppressive state (82).

Tregs and exhausted CD8 T cells shape the adaptive immune layer of local escape. FOXP3 expression has been linked to invasion and immune escape in CCA models, and tumor-infiltrating lymphocyte studies show that lymphocyte composition is associated with clinical outcome (91, 92). Multimodal single-cell analysis refined this picture by defining hyperactivated Tregs and insufficient tumor-reactive CD8 T cell infiltration in intrahepatic CCA (42). The challenge is therefore not only to deplete Tregs or stimulate CD8 T cells, but to reorganize the spatial interface where Tregs, CAFs, myeloid cells and exhausted T cells interact.

Antigen presentation is an underappreciated determinant of local escape and lymphatic spread. Defective DC recruitment can prevent productive priming of tumor-reactive T cells even when tumor antigens are present. In intrahepatic CCA, DC and CD8 T cell infiltration are decreased in tumors with lymph node metastasis; mechanistically, tumor-intrinsic beta-catenin activation can suppress CXCL12, resulting in defective DC recruitment, while Flt3L plus poly(I:C) can mobilize conventional DCs and reduce lymph node metastasis in models (59). This iCCA study directly connects local immune architecture with nodal dissemination and provides one of the clearest disease-specific rationales for metastasis-intercepting immunotherapy.

Taken together, these findings suggest that, at least in iCCA, effective intervention may require reorganization of the spatial interface among Tregs, CAFs, myeloid cells and dysfunctional CD8 T cells rather than manipulation of T-cell abundance alone. The principal cellular states, spatial distributions and candidate assays across the invasive-front and local-escape ecosystems are summarized in **Table 2**.

**Table 2:** Cellular and stromal programs that may organize the CCA metastatic cascade.

Cellular or stromal program	Likely spatial enrichment	Metastasis-enabling function	Candidate biomarkers/assays	Refs
Malignant epithelial plasticity	Invasive front, lymphovascular interfaces and metastatic lesions	Partial EMT, survival under stress, immune evasion and organ adaptation	EPCAM/KRT with VIM, ZEB1/2, SNAI; scRNA-seq trajectory; spatial RNA/protein	(46, 93, 94)
POSTN+FAP+ CAFs	Invasive leading edge and desmoplastic stromal tracks	ECM remodeling, immune exclusion and tumor–stroma organization	POSTN, FAP, COL1A1, ACTA2; multiplex IF around tumor margin	(46, 49, 86)
HSC-derived myCAF/iCAF states	Primary tumor stroma; potentially perivascular or portal areas	HAS2-hyaluronan matrix and HGF-MET trophic support	HAS2, HGF, MET, PDGFR; single-cell CAF subtype annotation	(49, 77, 79)
LGALS1+ fibroblasts	iCCA stromal neighborhoods	Promotes proliferation and migration through adhesion and receptor programs	LGALS1; CCR2, ADAM15, beta-integrin-linked tumor signatures	(51)
SPP1+ TAMs	Invasive front, fibrotic TAM-CAF neighborhoods and possible metastatic niches	Candidate fibrotic and immunosuppressive myeloid state	SPP1 with CD68/CD163; tumor proximity; SPP1-CD44/integrin ligands	(44, 46, 86)
Immature neutrophils/granulocytic MDSCs	Invasive front and myeloid-rich niches; possibly PMNs	T-cell suppression, matrix remodeling and metastatic seeding	CXCL5, CXCR2, S100A8/9, MPO, NE; spatial neutrophil states	(87–90)
Tregs	Tumor-immune interfaces, nodal tolerance zones and metastatic niches	Suppression of DC/T-cell priming and local cytotoxicity	FOXP3, CTLA4, IL2RA, MEOX1 program; Treg-to-CD8 spatial ratios	(42, 43, 91)
Tumor-reactive CD8 T cells	Inflamed tumors; sometimes excluded from malignant nests	Protective when antigen-specific, cytotoxic and physically proximate to tumor cells	CD39, GZMB, PRF1, TCF7, PDCD1; TCR clonality plus spatial localization	(42, 74, 95)
Exhausted or bystander T cells	Tumor core or stromal margins depending on tumor architecture	May inflate immune-inflamed signatures without productive killing	PDCD1, LAG3, HAVCR2 with TCR clonality and tumor-cell distance metrics	(42, 74, 92)
DC and cDC1 programs	Tumor core, invasive front, draining nodes and tertiary lymphoid-like areas	Antigen presentation, cross-priming and nodal immune control	CLEC9A, XCR1, BATF3, CCR7, CXCL12 axis; DC-CD8 neighborhoods	(59–62)
Endothelial and lymphatic endothelial cells	Vascular/lymphatic entry sites and tumor-draining nodes	Vascular leak, lymphangiogenesis, immune-cell trafficking and tolerance	CD31, LYVE1, PROX1, D2-40; VEGF-C/D; lymphatic density	(56–58, 96–98)
Platelets and coagulation-associated cells	Early hematogenous transit and vascular arrest	Shielding circulating tumor cells and supporting initial seeding	Platelet-tumor aggregates; thromboinflammatory markers	(99, 100)
ECM and matrix-remodeling program	Desmoplastic core, invasive front and target-organ matrix	Mechanical guidance, integrin signaling, growth-factor retention and T-cell exclusion	Collagen alignment, LOX, hyaluronan, stiffness, second harmonic generation	(52, 53, 72, 80, 81)
EV-mediated intercellular communication	Tumor-CAF-TAM axis, blood, bile and target-organ niches	Carries miRNA, circRNA and proteins that modulate stromal and immune states	EV cargo panels; MISEV-compliant isolation; paired tissue-EV validation	(39, 55, 67–71)

This table is a concise evidence map rather than a list of uniformly established mechanisms. Rows described as “candidate,” “possible,” “inferred” or “requiring validation” are hypothesis-generating. Direct single-cell or spatial support for several programs—including POSTN+FAP+ CAFs, LGALS1+ fibroblasts, MEOX1-associated Tregs and DC deficits—derives predominantly from iCCA; their relevance to perihilar and distal CCA remains to be established.

## 7 Lymphovascular entry and the transition from local invasion to systemic education

Metastasis requires access to routes of dissemination. In CCA, lymphovascular invasion and lymph node metastasis are strong adverse prognostic factors, indicating that lymphatic and vascular interfaces are biologically meaningful. Lymphangiogenesis has been described in CCA and can be driven by tumor-stroma interactions, including platelet-derived growth factor-D-related activation of myofibroblasts and lymphatic programs (23, 56, 57). Perineural invasion, another common feature in biliary cancers, may also reflect crosstalk among tumor cells, stromal cells, nerves and immune mediators that facilitates local spread and pain, although its immune role in metastasis is less well defined (96).

Endothelial cells are not passive conduits. Tumor-associated blood and lymphatic endothelium regulate immune-cell trafficking, vascular permeability, intravasation and extravasation. VEGF and angiopoietin pathways can suppress DC maturation and T cell trafficking while promoting abnormal vasculature, and anti-angiogenic therapy can sometimes normalize vessels and improve immune access (97, 101). In gastrointestinal cancers, lymphatic endothelial cells can present antigens, express immune checkpoints and promote tolerance, implying that lymphatic routes may actively condition anti-tumor immunity during nodal spread (58, 98).

CCA studies provide proof-of-concept that EVs can mediate local tumor–stroma and tumor–myeloid communication. In one study, TAM-derived exosomal circ_0020256 was reported to promote CCA proliferation, migration and invasion through a candidate circ_0020256/miR-432-5p/E2F3 axis (55). A separate human iCCA study reported a candidate tumor EV miR-9-5p–IL-6 communication axis involving vascular CAFs (38). EV-focused reviews have described additional CCA-associated cargoes, but their roles in systemic education or organ-specific niche formation remain incompletely established (39).

**Figure 3:**
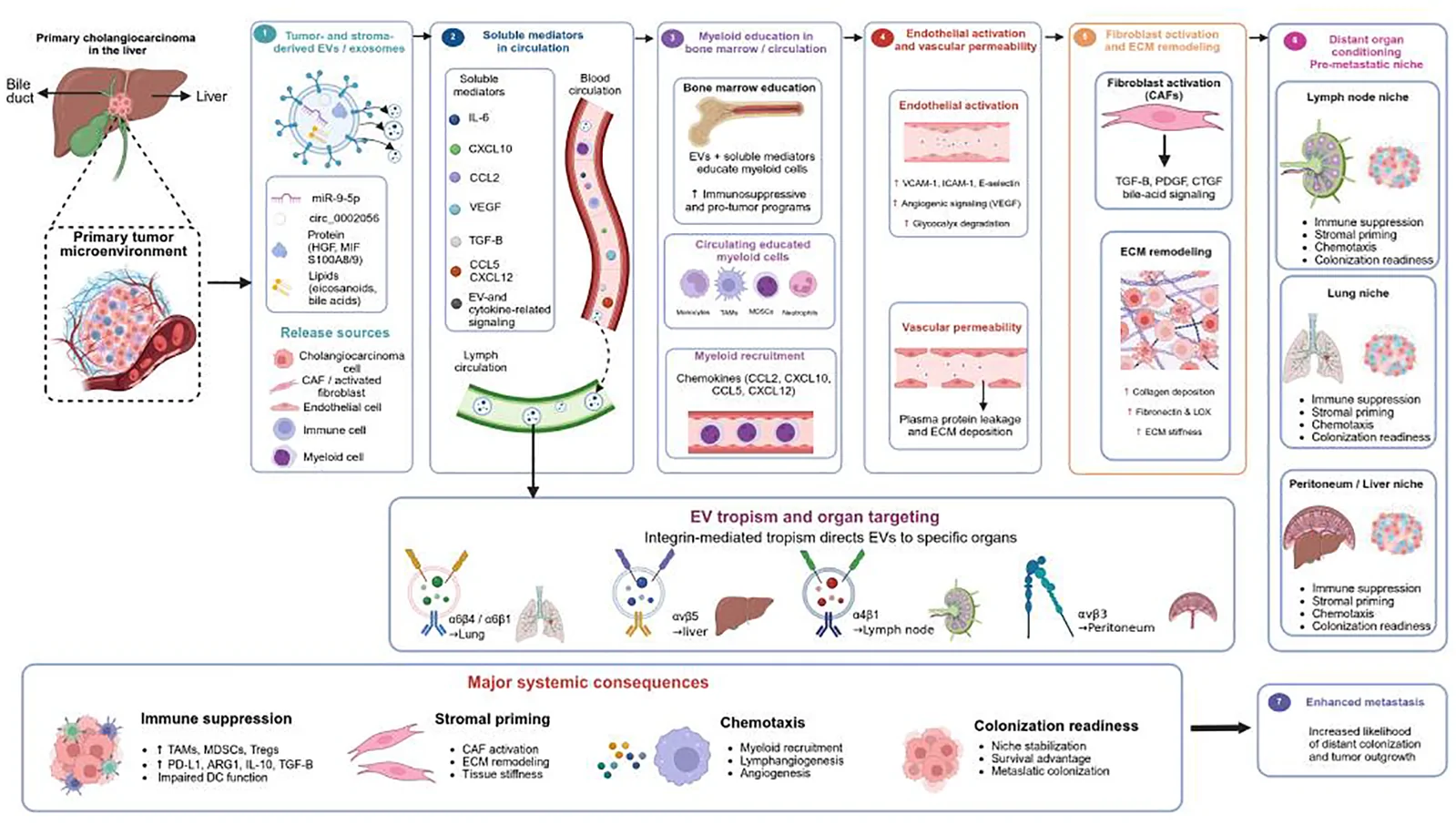
Proposed systemic education and pre-metastatic niche formation in cholangiocarcinoma. This model illustrates how the primary cholangiocarcinoma microenvironment may educate systemic and distant organ compartments before or during metastatic dissemination. CCA-specific EV evidence includes both subtype-unspecified studies and iCCA-derived observations, whereas organotropic pre-metastatic niche mechanisms remain largely inferred from other malignancies. Cholangiocarcinoma cells, activated CAFs, endothelial cells, immune cells and myeloid cells release extracellular vesicles and soluble mediators into the blood and lymphatic circulation. EV cargoes reported in individual CCA studies include miR-9-5p and circ_0020256, but their roles in systemic or organ-specific niche education remain unproven. In the proposed model, EVs and soluble mediators such as IL-6, CXCL10, CCL2, VEGF, TGF-β, CCL5 and CXCL12 may contribute to systemic communication and the development of immunosuppressive myeloid states. In the proposed model, endothelial activation, vascular permeability and glycocalyx remodeling could increase tissue accessibility for circulating tumor-derived factors and myeloid cells. Fibroblast activation and ECM remodeling, characterized by collagen deposition, fibronectin accumulation, LOX activity and increased matrix stiffness, could further contribute to permissive distant-organ states. Organotropic EV uptake and integrin-mediated homing may direct niche formation toward lymph node, lung and peritoneal/liver sites. The hypothesized systemic consequences include immune suppression, stromal priming, chemotaxis, angiogenesis and increased permissiveness to metastatic outgrowth. These distant-organ mechanisms are inferred mainly from adjacent cancers and have not been directly demonstrated before tumor-cell arrival in CCA. CAF, cancer-associated fibroblast; CCA, cholangiocarcinoma; CCL2/5, C-C motif chemokine ligands 2 and 5; circ_0020256, circular RNA 0020256; CTGF, connective tissue growth factor; CXCL10/12, C-X-C motif chemokine ligands 10 and 12; ECM, extracellular matrix; EV, extracellular vesicle; HGF, hepatocyte growth factor; IL-6, interleukin-6; LOX, lysyl oxidase; MDSC, myeloid-derived suppressor cell; MIF, macrophage migration inhibitory factor; miR-9-5p, microRNA-9-5p; PDGF, platelet-derived growth factor; S100A8/9, S100 calcium-binding proteins A8 and A9; TAM, tumor-associated macrophage; TGF-β, transforming growth factor beta; Treg, regulatory T cell; VCAM-1, vascular cell adhesion molecule 1; VEGF, vascular endothelial growth factor.

The available evidence raises the possibility that CCA dissemination is influenced by both cellular and acellular signals. Malignant cells enter lymphovascular routes, but EVs, cytokines, chemokines and matrix-remodeling enzymes may reach regional lymph nodes and distant organs earlier or in parallel. In other cancers, tumor EVs can carry integrins and other cargo that promote organotropic metastasis, while pancreatic cancer exosomes can educate liver Kupffer cells and hepatic stellate cells to form a fibrotic pre-metastatic niche (63, 64). These concepts should be tested directly in CCA using paired plasma EVs, bile-derived EVs, tumor tissue and target-organ samples. **Figure 3** presents a hypothesis-generating model linking local invasive-front biology with possible systemic and distant-organ conditioning.

## 8 Pre-metastatic niches: what is known, what is inferred and what remains to be proven in CCA

The pre-metastatic niche (PMN) concept originated from the observation that tumor-derived signals can recruit VEGFR1-positive bone marrow-derived cells to future metastatic sites before tumor cell arrival (65). Subsequent work established that PMNs are organ-specific ecosystems involving EVs, tumor-secreted factors, vascular permeability, ECM remodeling, fibroblast activation, resident macrophages, neutrophils and immune suppression (15, 66). This concept is now well developed in melanoma, breast, lung, colorectal and pancreatic cancers, but remains underexplored in CCA.

A careful CCA review should distinguish CCA-specific observations relevant to PMN biology from direct evidence of pre-arrival niche formation. Current CCA data include EV-mediated tumor–stroma–myeloid communication, invasion-associated CAF and myeloid programs, remodeling of established lymph-node metastases and organ-specific effects of decellularized ECM. These findings provide biological plausibility but do not demonstrate that CCA-derived factors condition the lung, peritoneum, liver or bone before tumor-cell arrival. Organ-specific PMNs in CCA should therefore be regarded as hypothesis-generating until validated through longitudinal sampling or paired analysis of primary tumors, circulation and initially tumor-free target organs.

EV biology provides the strongest bridge. Standard EV biology has defined exosomes and related small EVs as carriers of proteins, lipids, DNA and RNA that mediate intercellular communication (67, 68). The MISEV guidelines have improved rigor in EV nomenclature and characterization, which is essential for translating PMN studies into reproducible biomarkers (69). In cancer, EVs can remodel distant stromal and immune compartments, promote vascular leak, transfer oncogenic cargo and influence organotropism (70, 71). CCA EV studies should adopt these standards and move beyond *in vitro* migration assays toward organ-specific functional models.

Myeloid education is the second major PMN module. Tumor-derived factors can mobilize neutrophils, monocytes and MDSCs that suppress T cell activity and support metastatic seeding. In pancreatic cancer, exosome-driven liver PMN formation involves Kupffer-cell education, TGF-beta signaling and fibronectin deposition (64). In colorectal cancer and other gastrointestinal malignancies, neutrophils, macrophages and platelets contribute to circulating tumor-cell survival, extravasation and niche formation (99, 100). In CCA, the reported TAM–EV, CXCL5–neutrophil and bile acid–CAF–neutrophil pathways raise the possibility that myeloid programs contribute to systemic conditioning; however, these primarily local or primary-tumor observations do not demonstrate myeloid remodeling in distant organs before tumor-cell arrival (55, 82, 87). **Figure 3** distinguishes CCA-supported EV mechanisms from PMN mechanisms inferred from adjacent cancer biology, and **Table 2** identifies the myeloid and stromal programs that should be prioritized for validation.

ECM remodeling is the third PMN module. Lysyl oxidase-mediated collagen crosslinking, fibronectin deposition and periostin-rich matrices can make distant organs permissive for disseminated tumor cells in several cancers (72, 80). Because CCA is highly desmoplastic, it is plausible that matrix-derived signals and CAF-like stromal activation are central to organotropism. The most direct CCA evidence comes from organoid-ECM modeling, where patient-derived CCA organoids cultured in decellularized human lung and lymph node matrices showed organ-specific changes in EMT, stemness and migration/proliferation-related genes (73). These data demonstrate that organ-derived ECM can alter CCA cell states after tumor cells encounter the matrix, supporting organ-specific selection during metastatic colonization. They do not demonstrate that CCA remodels the lung or lymph-node ECM before tumor-cell arrival.

## 9 Organotropic niches in CCA metastasis

Regional lymph nodes are clinically important metastatic sites in CCA, although the detailed prognostic and mechanistic evidence reviewed here derives predominantly from iCCA. In iCCA, the number and station of metastatic lymph nodes after curative-intent resection influence prognosis and are relevant to staging and therapeutic decision-making (102). Beyond staging, lymph nodes are immune organs in which antigen presentation, tolerance, T-cell priming, Treg enrichment and macrophage remodeling can either prevent or enable metastatic growth. This makes nodal disease an important setting for metastasis-intercepting immunotherapy.

A single-cell atlas of iCCA lymph-node metastases reported extensive remodeling of stromal and immune compartments and identified an atlas-derived candidate interaction between SAA1-positive tumor cells and CD36-positive macrophages (75). An independent iCCA study found reduced DC and CD8 T-cell infiltration in tumors with lymph-node metastasis and showed that restoration of DC recruitment and activation could reduce nodal metastasis in experimental models (59). Together, these iCCA studies support a candidate nodal ecosystem characterized by impaired antigen presentation, macrophage adaptation and T-cell dysfunction rather than passive tumor-cell lodging alone.

The lung is another common distant site for intrahepatic CCA metastasis. Retrospective analysis of 370 patients with intrahepatic CCA reported lung, peritoneal and bone metastases as important distant sites, with metastatic location carrying clinical relevance (103). Lung PMNs in other cancers are shaped by vascular permeability, S100 proteins, neutrophils, fibroblasts, platelets and ECM changes (104, 105). Direct evidence for lung pre-metastatic niche formation remains limited across CCA subtypes. Nevertheless, a CCA organoid model showed that decellularized lung ECM can alter tumor-cell phenotypes, supporting the possibility of organ-specific matrix adaptation (73). Because this model begins after tumor cells are introduced into lung-derived ECM, it should not be interpreted as evidence that CCA establishes a lung PMN before tumor-cell arrival.

The liver and peritoneum are biologically distinctive target sites. Intrahepatic metastasis and satellite lesions occur within a liver microenvironment containing sinusoidal endothelium, Kupffer cells, hepatic stellate cells, bile ducts, portal fibroblasts and chronic injury-associated immune cells. Peritoneal dissemination, in contrast, involves mesothelial cells, omental adipose tissue, peritoneal macrophages, ascitic cytokines and fibrin-rich matrices. Studies in other gastrointestinal cancers show that peritoneal niches can be strongly influenced by macrophages, neutrophils, fibroblasts and adipocyte-derived factors (106, 107). CCA-specific peritoneal immune niche studies are scarce and should be prioritized. Accordingly, the proposed mesothelial, myeloid and matrix features should be regarded as candidate determinants of peritoneal colonization rather than demonstrated components of a CCA pre-metastatic niche.

Bone metastasis is a clinically recognized but immunologically understudied distant site in iCCA. Bone is an immune and stromal organ where osteoclasts, osteoblasts, marrow adipocytes, endothelial cells and hematopoietic niches interact with disseminated tumor cells. General metastasis literature shows that tumor cells exploit RANKL, CXCL12, TGF-beta and osteoclast-mediated matrix release to colonize bone (108, 109). Whether iCCA uses similar bone-colonization programs remains unclear, but its occurrence in clinical cohorts supports investigation of bone-specific immune–stromal adaptation (103).

**Figure 4:**
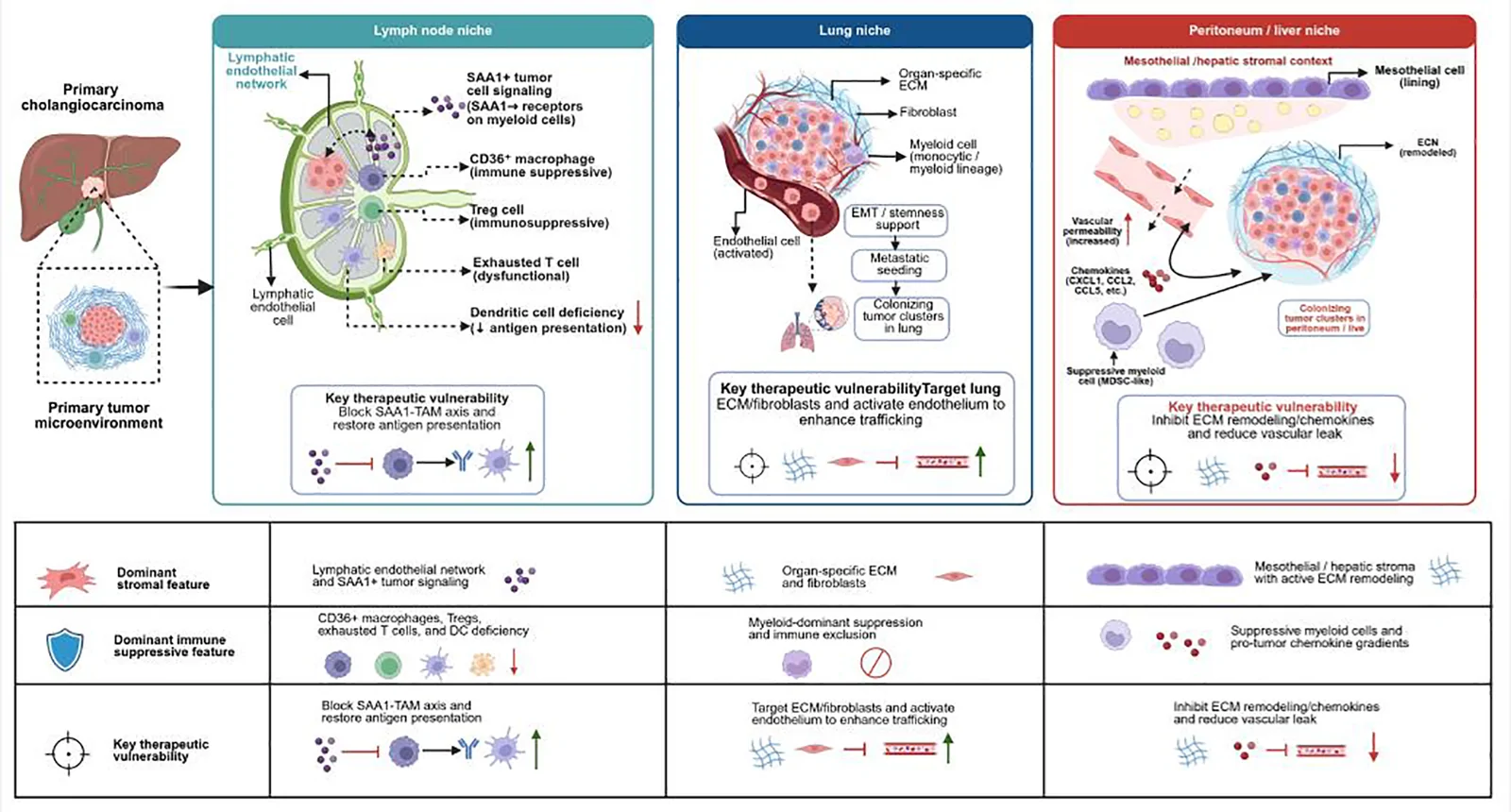
Organotropic immune niches in cholangiocarcinoma metastasis. This schematic compares three representative organotropic immune niches that may support cholangiocarcinoma metastasis: lymph node, lung and peritoneum/liver. The clinical distribution of distant metastases is supported by iCCA data, whereas the SAA1–CD36 nodal crosstalk signal derives from a single iCCA atlas and remains an unvalidated candidate. The broader organ-specific immune architectures depicted here are hypothesis-generating for CCA. In the proposed lymph node niche, lymphatic endothelial and stromal networks may interact with SAA1-positive tumor signaling, CD36-positive macrophages, Tregs, exhausted T cells and dendritic-cell deficiency, potentially generating an immune-tolerant environment with impaired antigen presentation. In the proposed lung niche, organ-specific ECM, lung fibroblasts, activated endothelial cells and monocyte-lineage myeloid cells may support EMT/stemness-associated adaptation, metastatic seeding and colonizing tumor clusters. In the proposed peritoneal or liver niche, mesothelial or hepatic stromal contexts, vascular permeability, chemokine gradients and active ECM remodeling may promote suppressive myeloid-cell recruitment and tumor cluster colonization. These descriptions concern candidate colonization ecosystems and should not be interpreted as direct evidence of pre-metastatic conditioning. The lower comparison panel summarizes dominant stromal features, dominant immune-suppressive features and key therapeutic vulnerabilities for each niche. Potential interception strategies include testing whether candidate SAA1–CD36 macrophage crosstalk can be disrupted and restoring antigen presentation in lymph nodes, targeting lung ECM/fibroblasts and endothelial trafficking in lung metastasis, and inhibiting ECM remodeling, chemokines and vascular leak in peritoneal or liver colonization. CAF, cancer-associated fibroblast; CCA, cholangiocarcinoma; CCL2/5, C-C motif chemokine ligands 2 and 5; CD36, cluster of differentiation 36; CXCL1, C-X-C motif chemokine ligand 1; DC, dendritic cell; ECM, extracellular matrix; EMT, epithelial-mesenchymal transition; MDSC, myeloid-derived suppressor cell; SAA1, serum amyloid A1; TAM, tumor-associated macrophage; Treg, regulatory T cell.

A useful way to frame organotropism in the manuscript is therefore to ask which target-organ features are permissive: nodal tolerance and antigen presentation defects, lung ECM and vascular beds, liver fibrosis and sinusoidal macrophages, peritoneal inflammation and mesothelial barriers, or bone marrow niches. This framing avoids assuming a single universal metastatic niche and instead invites comparative spatial studies across organs. **Figure 4** illustrates this organotropic framework, whereas **Table 3** summarizes niche-specific clinical and model evidence, candidate permissive features and experimental priorities, while distinguishing direct CCA/iCCA observations from cross-cancer inference and unproven pre-metastatic niche mechanisms.

**Table 3:** Organotropic niches and testable hypotheses for CCA metastasis.

Target-organ niche	Clinical/model evidence	Candidate permissive features or inferred mechanisms	Biomarker or experiment to prioritize	Refs
Regional lymph node	Nodal metastasis burden and station predict outcome after iCCA resection; scRNA-seq identifies nodal microenvironment remodeling.	DC scarcity, T-cell dysfunction and candidate SAA1+ tumor cell–CD36+ macrophage crosstalk	Paired primary-LN spatial omics; SAA1-CD36 proximity; DC and CD8 neighborhoods	(59, 75, 102)
Draining lymphatic route	Lymphatic vessel density and lymphangiogenesis correlate with nodal spread and prognosis in biliary tumors.	VEGF-C/D signaling, tolerance mediated by lymphatic endothelial cells and altered immune trafficking	D2-40/LYVE1/PROX1 quantification at invasive front and tumor-draining nodes	(56–58)
Lung—PMN hypothesis-generating	Distant lung metastases are clinically observed; CCA organoids show organ-specific responses to decellularized lung ECM.	Candidate ECM-mechanical, endothelial and alveolar-myeloid features; neutrophil-rich PMN modules inferred from other cancers	Lung ECM-organoid-immune co-culture; lung metastasis biopsy spatial profiling	(73, 103, 110)
Intrahepatic satellite/liver parenchyma—niche mechanisms inferred	iCCA commonly recurs within liver and may arise in chronically inflamed or fibrotic liver.	Fibrotic matrix, sinusoidal endothelium, Kupffer-like macrophages and bile-acid/inflammatory signaling	Compare tumor core, margin and adjacent liver; include cholestasis and fibrosis covariates	(1, 23, 41)
Peritoneum—PMN hypothesis-generating	Peritoneal spread occurs in advanced iCCA clinical series.	Candidate mesothelial, macrophage-rich and matrix-attachment features inferred from established peritoneal disease and adjacent gastrointestinal cancers; pre-arrival conditioning unproven in CCA	Peritoneal lesion biopsies; peritoneal fluid EV and myeloid profiling	(103, 106, 107)
Bone—niche mechanisms inferred	Bone metastasis is a recognized distant site in iCCA cohorts.	Marrow stromal niches, osteoclast/osteoblast crosstalk, dormancy and myeloid suppression	Bone metastasis transcriptomics; marrow immune profiling; skeletal-event-associated biomarkers	(103, 108, 109)
Systemic PMN state—cross-cancer inference	Canonical non-CCA studies show that tumor-secreted factors and EVs can recruit marrow-derived cells before tumor arrival.	VEGFR1+ progenitors, LOX-mediated ECM remodeling, exosome integrins, endothelial leak and neutrophils; all remain unvalidated as CCA PMN mechanisms	Longitudinal EV/myeloid panels in high-risk resected CCA; organ-specific relapse correlation	(15, 16, 63–66)
Metastatic cold ecosystem—hypothesis-generating	Metastatic lesions may retain or acquire organ-specific immune exclusion despite immune-inflamed primary signatures.	T-cell exclusion, antigen-presentation defects, Treg/TAM dominance and ECM barriers	Compare primary vs metastasis biopsies for CD8-to-tumor distance, DC abundance and CAF/TAM neighborhoods	(111–113)
Therapy-conditioned niche—mixed evidence	Chemotherapy, antiangiogenic therapy and ICI can remodel immune access and selection pressures.	Immunogenic cell death, vascular normalization, myeloid remodeling and resistant stromal states	On-treatment biopsy or liquid biopsy; spatial pharmacodynamic endpoints	(9, 10, 83, 94, 97)

MFS, metastasis-free survival. The evidence column distinguishes direct CCA evidence from inference based on adjacent-field biology. No organ-specific CCA PMN has yet been directly demonstrated before tumor-cell arrival; rows labeled “hypothesis-generating” or “inferred” should not be interpreted as established CCA PMN mechanisms.

## 10 Metastatic colonization and immune escape: from cold tumors to cold ecosystems

Once disseminated CCA cells reach a target organ, colonization requires survival, dormancy escape, growth and immune evasion. General metastasis biology shows that many disseminated tumor cells die or remain dormant, and successful colonization depends on reciprocal adaptation between cancer cells and host tissue (13, 14). Immune surveillance can eliminate disseminated cells, but chronic inflammation, myeloid suppression and stromal barriers can convert potential immune control into tolerance (111, 112).

In CCA, metastatic colonization likely involves malignant epithelial plasticity. EMT and partial EMT programs can promote invasion, stemness, resistance to apoptosis and immune evasion. Reviews of EMT in CCA have emphasized interactions among TGF-beta, Notch, Wnt/beta-catenin, Hedgehog, hypoxia and inflammatory pathways (93, 114). In iCCA, recent single-cell metastasis studies provide a route to identify metastasis-associated epithelial states rather than treating EMT as a binary process (76).

Immune escape at metastatic sites may differ from escape in the primary tumor. For example, a nodal metastasis may develop in an immune organ rich in lymphocytes but dominated by tolerance, exhausted T cells and macrophage remodeling, whereas a lung metastasis may be shaped by alveolar macrophages, neutrophils and ECM-driven mechanical signals. Bulk PD-L1 expression alone cannot resolve these organ-specific immune states. Microsatellite instability-high/mismatch repair-deficient (MSI-H/dMMR) status identifies an uncommon but clinically relevant tissue-agnostic subgroup (115, 116). CCA-specific data further associate MSI-H with higher tumor mutational burden, greater PD-L1 positivity and improved outcomes after PD-1 inhibitor-based therapy (117). Tumor mutational burden-high (TMB-H) may also enrich for immune-checkpoint inhibitor benefit in advanced biliary tract cancer, but neither genomic biomarker captures the spatial organization of antigen presentation, T-cell exclusion, CAF–myeloid barriers or organ-specific metastatic niches (118). MSI-H/dMMR and TMB-H should therefore complement rather than replace spatial profiling.

The term cold tumor should therefore be expanded to cold ecosystem. A tumor can appear immune inflamed by transcriptome yet remain spatially nonproductive if CD8 T cells are trapped outside malignant nests, if DCs are absent, if TAMs and CAFs dominate the invasive front, or if Tregs occupy key tumor-immune interfaces. Conversely, a low-cellularity metastasis may be vulnerable if antigen presentation is intact and suppressive stromal barriers are weak. Spatial assessment is needed to distinguish these states and to select rational combinations. **Figure 4** provides an organ-level representation of how these cold ecosystems may differ across lymph node, lung and peritoneal/liver niches.

## 11 Therapeutic implications and intervention windows

Operationally, this framework encompasses intervention at the invasive front, during lymphovascular dissemination and within emerging or established metastatic niches. In CCA, this concept is clinically attractive for high-risk resected patients, borderline resectable disease, locally advanced tumors with threatened vascular or nodal spread and molecularly defined subgroups with high recurrence risk. It is also relevant for advanced disease because established metastases may still depend on niche maintenance.

The first intervention window is the invasive front. CAF-directed strategies should prioritize state-specific reprogramming. Candidate mediators for experimental testing include HGF–MET, HAS2–hyaluronan, LGALS1, PDGF-related recruitment and the bile acid–GPBAR1–CXCL10 axis. Indiscriminate CAF depletion should be avoided because CAF functions can be either tumor-promoting or tumor-restraining depending on cellular state and tissue context (49–51, 82, 119).

The second window is myeloid reprogramming. Candidate approaches include CSF1R inhibition, CCR2/CCR5 blockade, CXCR2 blockade, CD40 agonism and PI3K-gamma inhibition. Their use should be guided by spatial evidence of TAM-, neutrophil- or granulocytic MDSC-dominant suppression, because most of these strategies remain preclinical or have been developed primarily in other malignancies (83, 120–123).

The third window is DC restoration and lymph-node immune protection. The available iCCA evidence provides a rationale for testing Flt3L/poly(I:C), CD40, STING or TLR agonists, tumor vaccines and antigen-releasing therapies in perioperative or nodal-risk settings. DC recruitment, CD8 T-cell priming and nodal immune architecture should be incorporated as pharmacodynamic endpoints in such studies (59–62).

The fourth window is EV and PMN disruption. Because CCA EV studies have identified tumor-CAF and TAM-tumor communication, EV release, uptake or cargo function could theoretically be targeted, although clinical translation remains early (39, 55). More immediately, EV cargoes may serve as biomarkers of niche-forming activity, especially when combined with circulating tumor DNA, inflammatory markers and spatial tissue signatures. Lessons from PMN biology caution that single EV cargoes are unlikely to be sufficient; panels capturing myeloid recruitment, ECM remodeling and endothelial activation may perform better (67, 70).

**Figure 5:**
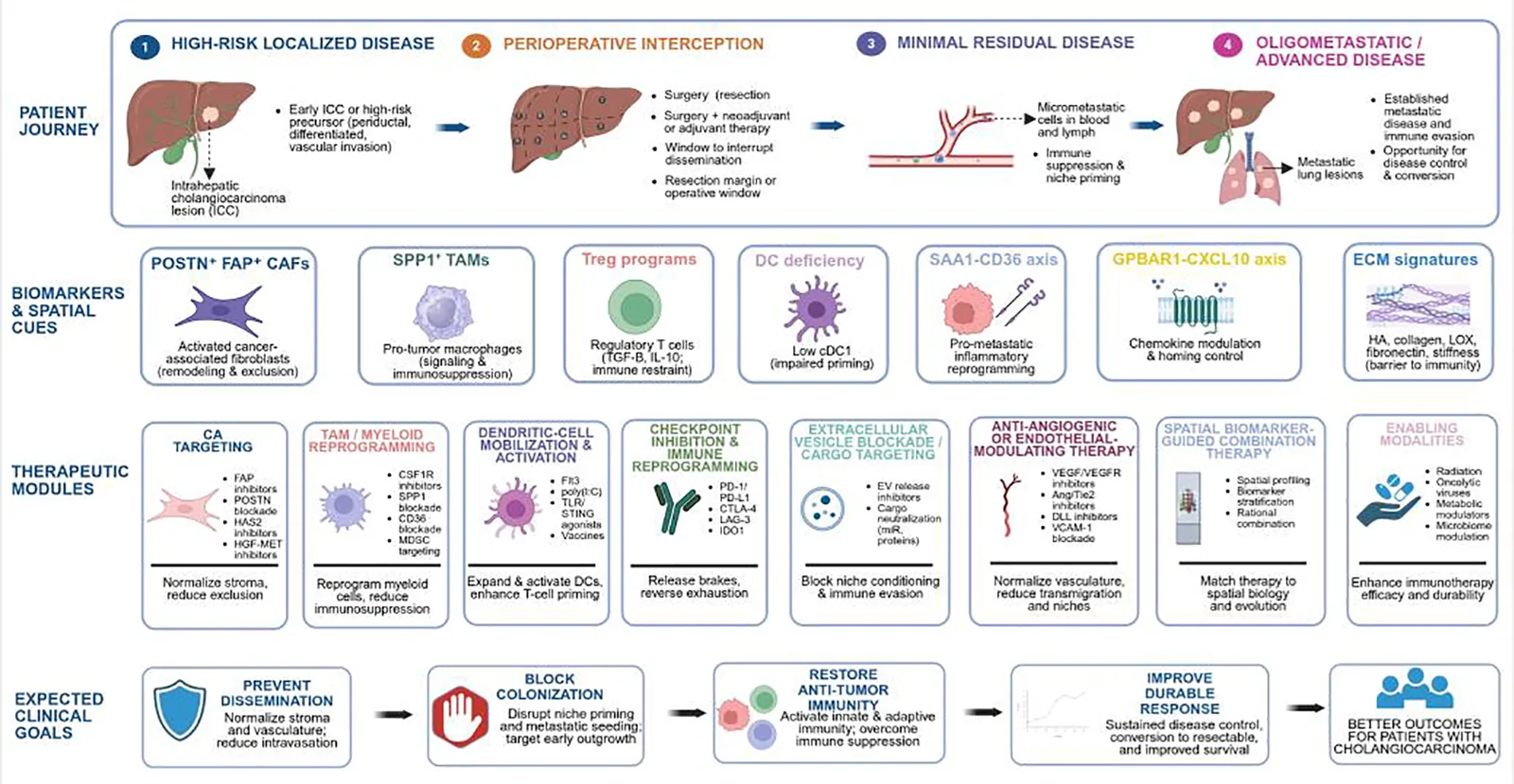
Metastasis-intercepting immunotherapy for cholangiocarcinoma. This roadmap integrates disease stage, spatial biomarkers, therapeutic modules and expected clinical goals for metastasis-intercepting immunotherapy in cholangiocarcinoma. Evidence maturity differs across the roadmap: immune-checkpoint inhibitor plus gemcitabine–cisplatin combinations constitute an established first-line clinical backbone for advanced biliary tract cancer, whereas the spatial biomarkers and niche-directed interception modules shown are translational, preclinical or hypothesis-generating rather than validated metastasis-interception strategies. Along the patient journey, high-risk localized disease, perioperative treatment windows, minimal residual disease and oligometastatic or advanced disease represent distinct opportunities to interrupt dissemination and metastatic outgrowth. Candidate spatial biomarkers include POSTN-positive/FAP-positive CAFs, SPP1-positive TAMs, Treg programs, dendritic-cell deficiency, candidate SAA1–CD36 crosstalk, candidate GPBAR1–CXCL10 signaling and ECM signatures such as hyaluronan, collagen, LOX, fibronectin and tissue stiffness. Among these, GPBAR1–CXCL10 signaling and SAA1–CD36 crosstalk each derive from a single recent CCA/iCCA study and have not been independently validated as selection biomarkers. Leading-edge POSTN/FAP-rich states, MEOX1-associated Tregs and DC deficiency also require validation across anatomical CCA subtypes. These investigational biomarkers may guide candidate therapeutic modules, including CAF targeting, TAM/myeloid reprogramming, dendritic-cell mobilization and activation, extracellular vesicle blockade or cargo targeting, anti-angiogenic or endothelial-modulating therapy, spatial biomarker-guided combination therapy and enabling modalities such as radiation, oncolytic viruses, metabolic modulation or microbiome modulation. Checkpoint inhibition has clinical support in defined systemic-treatment combinations, whereas the niche-directed applications proposed here require prospective validation. The expected clinical goals are to prevent dissemination, block metastatic colonization, restore antitumor immunity and improve durable disease control, conversion to resectability and survival. Ang/Tie2, angiopoietin/Tie2 receptor signaling; CAF, cancer-associated fibroblast; cDC1, type 1 conventional dendritic cell; CCA, cholangiocarcinoma; CSF1R, colony-stimulating factor 1 receptor; CTLA-4, cytotoxic T-lymphocyte-associated protein 4; CXCL10, C-X-C motif chemokine ligand 10; DC, dendritic cell; DLL4, delta-like ligand 4; ECM, extracellular matrix; EV, extracellular vesicle; FAP, fibroblast activation protein; Flt3L, Fms-like tyrosine kinase 3 ligand; GPBAR1, G protein-coupled bile acid receptor 1; HA, hyaluronan; HAS2, hyaluronan synthase 2; HGF, hepatocyte growth factor; iCCA, intrahepatic cholangiocarcinoma; IDO1, indoleamine 2,3-dioxygenase 1; LAG-3, lymphocyte activation gene 3; LOX, lysyl oxidase; MRD, minimal residual disease; PD-1, programmed cell death protein 1; PD-L1, programmed death-ligand 1; poly(I:C), polyinosinic-polycytidylic acid; POSTN, periostin; SAA1, serum amyloid A1; SPP1, secreted phosphoprotein 1; STING, stimulator of interferon genes; TAM, tumor-associated macrophage; TLR, Toll-like receptor; Treg, regulatory T cell; VCAM-1, vascular cell adhesion molecule 1; VEGF, vascular endothelial growth factor; VEGFR, vascular endothelial growth factor receptor.

The fifth window is rational combination with immune checkpoint blockade. TOPAZ-1 and KEYNOTE-966 demonstrate that checkpoint inhibition can add benefit to chemotherapy in advanced biliary tract cancer, but they do not define which spatial immune ecosystems are most responsive (9, 10). Future trials should integrate pre-treatment biopsies, on-treatment biopsies when feasible, spatial profiling of the invasive front, circulating EV/myeloid markers and paired analysis of metastatic sites. Combinations should be selected according to dominant resistance circuits: CAF-rich T cell exclusion may call for stromal reprogramming, myeloid-rich niches for TAM or neutrophil targeting, and DC-deficient tumors for antigen-presentation restoration. **Figure 5** illustrates the therapeutic roadmap, whereas **Table 4** consolidates individual pathways into broader intervention classes by treatment window and evidence maturity, separating trial-established backbones from translational, preclinical and hypothesis-generating spatial strategies.

**Table 4:** Metastasis-intercepting immunotherapy strategies and biomarker-guided trial design.

Intervention window	Target circuit/strategy	Rationale for metastasis interception	Potential combinations and patient setting	Biomarkers, caveats and evidence maturity
Baseline spatial triage	mIF/spatial transcriptomics of tumor core, invasive front, lymphatics and nodes	Identifies whether the dominant barrier is stromal exclusion, myeloid suppression, DC deficiency or Treg dominance.	High-risk resectable or locally advanced CCA before systemic therapy.	Translational: Immature field; needs standardized sampling and region annotation (124–129).
CAF-state reprogramming	HAS2-hyaluronan, HGF-MET, LGALS1 and PDGF-related CAF recruitment	CAF tracks and soluble programs may guide invasion and exclude T cells.	ICI/chemotherapy with CAF mediator blockade in CAF-rich tumors.	Preclinical: Avoid indiscriminate CAF depletion; CAF functions can be restraining or promoting (49–51, 119).
TAM reprogramming	CSF1R, CCR2/CCR5, PI3K-γ, SPP1-related macrophage circuits and candidate SAA1–CD36 nodal crosstalk	Reverses myeloid-driven matrix remodeling and T-cell suppression and tests whether nodal macrophage adaptation contributes to immune tolerance.	ICI or chemotherapy plus macrophage reprogramming in invasive-front TAM-high tumors; exploratory perioperative testing in high LNM-risk iCCA.	Preclinical: CCA-specific macrophage states require spatial biomarker selection; SAA1–CD36 crosstalk derives from a single nodal atlas and lacks functional and druggability validation (45, 54, 75, 120, 121).
Neutrophil/gMDSC targeting	CXCL5/CXCR2, the candidate GPBAR1–CXCL10-associated immature-neutrophil program and NET-related pathways	Reduces neutrophil-mediated invasion and immune suppression, including a candidate cholestatic CAF-associated state.	ICI plus CXCR2-axis, NET-targeting or pathway-guided modulation in neutrophil-high tumors.	Preclinical: CCA evidence supports CXCL5 and a single-study candidate GPBAR1–CXCL10 program; broader cancer evidence supports CXCR2- and NET-directed strategies. Clinical validation is lacking (82, 87, 89, 90, 122, 123).
DC restoration and nodal protection	Flt3L/poly(I:C), CD40, TLR or STING activation; beta-catenin-CXCL12-DC axis	Restores antigen presentation and nodal immune control.	Neoadjuvant/perioperative vaccine, radiation or ICI combinations.	Preclinical: Mechanistic iCCA model supports cDC mobilization; toxicity and timing require study (59–62).
Treg modulation	Treg depletion or functional attenuation; CTLA4-related strategies	Reduces suppressive interface when Tregs dominate tumor-immune contacts.	Selected use with ICI or vaccines in Treg-high spatial niches.	Preclinical: Risk of autoimmunity; requires tumor-localized pharmacodynamic biomarkers (42, 43).
EV/PMN disruption	EV biogenesis, uptake, integrin-mediated organotropism and cargo-defined PMN signals	May interrupt long-distance communication before niches become permissive.	High-risk minimal residual disease monitoring; exploratory interventional trials.	Hypothesis-generating: CCA EV evidence exists, but organ-specific PMN proof is incomplete (39, 55, 63–71).
Lymphangiogenesis and vascular normalization	VEGF-C/D, VEGF-A and vessel normalization strategies	May reduce lymphatic dissemination and improve immune-cell access.	Antiangiogenic or lymphangiogenic modulation with ICI/chemotherapy.	Preclinical: Need distinguish improved trafficking from enhanced dissemination (56, 57, 97, 101).
ECM remodeling	LOX, collagen alignment, hyaluronan and matrix stiffness	Reduces physical exclusion and organ-specific mechanical selection.	Stromal therapy plus ICI or cellular therapy in desmoplastic tumors.	Preclinical: Matrix has context-dependent roles; spatial biomechanics endpoints needed (52, 53, 72, 80, 81).
Checkpoint plus chemotherapy backbone	Durvalumab or pembrolizumab with gemcitabine-cisplatin	Current first-line immunotherapy base that may be improved by spatial selection.	Advanced disease; potential perioperative adaptation in trials.	Clinical: Trial-proven survival benefit but unselected response heterogeneity (9, 10).
Cellular therapy with niche engineering	CAR-T/CAR-NK/gamma-delta T cells plus stromal or myeloid modulation	Engineered cells require trafficking through CAF/ECM and survival in suppressive niches.	Antigen-selected tumors; combine with CAF/TAM or vascular normalization strategies.	Early clinical: CCA case-level signals; solid-tumor trafficking remains limiting (130–134).
Vaccine and antigen-release strategies	Personalized peptides, DC activation, radiation/chemo-induced antigen release	Could convert MRD and nodal niches into productive priming sites.	Perioperative MRD setting with ctDNA/EV monitoring and ICI.	Early clinical: CCA proof-of-concept exists; immune-spatial endpoints should be embedded (59–62, 135).
Adaptive response monitoring	ctDNA clearance, EV cargo shifts, repeat spatial biopsies and organ-specific MFS	Tests whether therapy changes the niche before radiographic progression.	All stages, especially MRD and neoadjuvant windows.	Translational: Requires prospective validation and harmonized sample handling (67–71, 128, 129).

This table prioritizes therapeutic logic rather than listing approved indications. Most strategies remain preclinical or hypothesis-generating in CCA and should be tested with spatial pharmacodynamic endpoints.

## 12 Biomarkers and trial design for a spatial metastasis framework

Spatial biomarkers should capture position, cell state and intercellular organization. Candidate readouts can be grouped into four functional categories: stromal exclusion, represented by invasive-front POSTN-positive FAP-positive CAF density and ECM architecture; myeloid suppression, represented by SPP1-positive TAM proximity, immature neutrophil states and candidate SAA1-positive tumor cell–CD36-positive macrophage neighborhoods; defective antigen presentation, represented by DC scarcity; and adaptive immune dysfunction, represented by MEOX1-associated Tregs and CD8 T-cell exclusion. These readouts remain investigational and require prospective validation in anatomically annotated CCA cohorts. Most of these candidates are currently supported predominantly by iCCA cohorts or models, and their applicability to perihilar and distal CCA remains uncertain.

Quantitative support for these candidate biomarkers remains heterogeneous and is generally insufficient to define clinically actionable cutoffs. The leading-edge atlas profiled 25 tissue specimens from nine patients with iCCA, including seven tumor-core, nine leading-edge and nine adjacent non-neoplastic samples, with spatial transcriptomics performed on five leading-edge specimens (46). The lymph-node atlas analyzed 123,127 cells from metastatic lymph nodes, non-metastatic lymph nodes, primary tumors and adjacent liver tissue; in an independent iCCA cohort, SAA1 and CD36 expression showed a modest positive correlation (r = 0.38, P < 0.01) (75). For antigen-presentation biomarkers, reduced DC infiltration was evaluated across a 14-sample single-cell cohort, a 244-patient bulk-transcriptomic cohort and a 219-patient tissue-microarray cohort; higher intratumoral DC abundance was associated with longer overall and recurrence-free survival in a 25-patient discovery cohort (both P < 0.05) (59). By contrast, the MEOX1-associated Treg program was derived from 12 matched tumor and peritumoral samples from six patients and was associated with poor prognosis, but no clinically validated threshold was established (42). These results should therefore be regarded as study-level quantitative benchmarks rather than ready-to-use biomarker cutoffs.

Microbial biomarkers may provide an additional compartment-specific layer. Recent studies have identified CCA-associated microbial patterns in bile and intratumoral tissue, as well as multicohort fecal metagenomic signatures, although their causal and immunotherapy-predictive roles remain unproven (136, 137). Within a spatial framework, bile, tumor tissue and stool should be treated as biologically distinct sampling compartments. Biliary and intratumoral microbial features should be related to bile-acid exposure, epithelial proximity and local immune–stromal neighborhoods, whereas stool-derived signatures may serve as complementary non-invasive readouts. Future studies should collect matched stool, bile and tumor tissue where feasible and record antibiotic exposure, biliary drainage or stenting, cholangitis and anatomical subtype.

Modern technologies make these biomarkers feasible. Multiplex immunofluorescence, imaging mass cytometry and multiplexed ion beam imaging can quantify spatial neighborhoods at single-cell resolution (124, 125). Spatial transcriptomics can map gene expression across tissue compartments, and newer high-resolution platforms can integrate transcriptome, protein and morphology (126, 127). Single-cell RNA sequencing, single-cell ATAC sequencing and T cell receptor sequencing can define states and clonality, while paired spatial validation determines whether those states occupy metastasis-relevant locations (128, 129).

Trial design should match the biology. For resectable high-risk CCA, neoadjuvant or perioperative studies could sample primary tumor core, invasive front, regional lymph nodes and blood EVs before and after therapy. For locally advanced disease, paired biopsies can test whether combination therapy converts an excluded invasive front into an inflamed and antigen-presenting interface. For metastatic disease, sampling at least one metastatic lesion is crucial because organ-specific niches may differ from the primary tumor. Adaptive trial designs could assign patients to CAF, myeloid, DC or EV-directed combinations based on spatial signatures rather than anatomical subtype alone. **Table 4** provides a practical structure for matching biomarkers, interventions and pharmacodynamic endpoints.

Endpoints should also be reconsidered. Radiographic response is important but may not capture metastasis interception. Relevant endpoints include circulating tumor DNA clearance, reduction in lymphovascular invasion, decrease in viable tumor cells in lymph nodes, changes in spatial immune neighborhoods, recurrence-free survival after surgery and organ-specific metastasis-free survival. In window-of-opportunity studies, changes in DC infiltration, CD8 T cell proximity to tumor cells, TAM phenotype and CAF barrier architecture could provide early pharmacodynamic evidence.

The spatial model also changes how negative data should be interpreted. A biopsy lacking abundant CD8 T cells may reflect true immune desertification, but it may also miss an inflamed invasive front or a nodal immune reaction. Conversely, a tumor core rich in immune transcripts may still be functionally excluded if cytotoxic lymphocytes remain separated from malignant nests by CAF-rich ECM. For this reason, future CCA studies should report the region sampled, the distance between immune and malignant cells, and whether spatial neighborhoods are analyzed independently from the tumor core. This reporting standard would reduce the risk of treating discordant immune biomarkers as biological contradictions.

A second implication is that timing matters. The most clinically visible metastasis is a late product of earlier events: local invasion, lymphovascular access, systemic education, immune evasion and colonization. Intervening after a lung or peritoneal metastasis is radiographically established may require dismantling a mature organ-specific ecosystem, whereas perioperative or neoadjuvant intervention may only need to prevent that ecosystem from forming. This temporal logic supports trials in high-risk resectable or locally advanced CCA, where tissue access and recurrence endpoints make metastasis interception measurable.

The model further suggests that CCA research should avoid single-axis explanations. Bile acids, CAFs, TAMs, neutrophils, Tregs, DCs and EVs are not competing hypotheses; they are interacting layers of one network. In the proposed network model, a GPBAR1–CXCL10-high state, as reported in a single recent CCA study, may recruit immature neutrophils, but those neutrophils may be retained by CAF-derived ECM and reinforced by macrophage-derived mediators. A DC-deficient tumor may fail to prime T cells, but stromal exclusion can still prevent any primed T cells from entering the correct compartment. Mechanistic studies should therefore perturb circuits in combination and measure network rewiring.

Finally, the framework is intentionally testable. If the invasive front is a dissemination checkpoint, then front-specific signatures should outperform tumor-core signatures for predicting lymphovascular invasion, nodal metastasis or early recurrence. If EVs educate organotropic niches, then EV cargo should correlate with subsequent metastatic site and should alter target-organ stromal or immune states in functional assays. If nodal tolerance is active, then DC restoration or macrophage reprogramming should reduce nodal tumor burden and improve T cell priming. These hypotheses can be embedded into translational studies without requiring a complete redefinition of clinical practice; **Table 4** indicates how they can be converted into trial-ready biomarker and endpoint choices.

## 13 Transferable principles from adjacent fields and their limits in CCA

Evidence from other malignancies is most useful when it defines broad, testable principles rather than presumed CCA mechanisms. Four principles remain relevant to the present framework: organ-specific selection, functional CAF heterogeneity, T-cell quality and positioning, and matrix-mediated immune exclusion.

Organ-specific selection is a general principle of metastasis (138), and CCA clinical patterns, single-cell analysis of iCCA lymph-node metastases and organoid responses to lung and lymph-node ECM support a role for target-organ context (73, 75, 103). However, pre-arrival niche formation in CCA remains unproven. CCA studies also identify distinct hepatic stellate cell-derived myofibroblastic and inflammatory CAF programs and LGALS1-positive fibroblasts (49, 51), supporting state-selective intervention rather than indiscriminate CAF depletion (50), although their causal roles in dissemination and relevance to perihilar and distal CCA remain uncertain.

Similarly, iCCA single-cell and spatial studies suggest that tumor-reactive CD8 T-cell state, Treg activity and physical position may be more informative than bulk lymphocyte abundance (42, 46), but prospective predictive validation is lacking. POSTN-positive FAP-positive fibroblast neighborhoods and matrix-remodeling programs at invasion-relevant interfaces support a candidate stromal-exclusion model (46, 49); nevertheless, no CCA study has shown that matrix-directed treatment restores productive T-cell access or prevents metastasis, and broad stromal depletion may remove tumor-restraining functions (50).

Together, these principles support comparative sampling of primary tumors, invasive fronts, regional lymph nodes and distant metastases, combined with spatial profiling and functional perturbation. Until such studies are available, they should be treated as hypotheses with differing levels of CCA-specific support.

## 14 Future directions

First, the field needs paired spatial atlases. Ideal cohorts would include tumor core, invasive front, adjacent liver or periductal tissue, draining lymph nodes, plasma and bile EVs, and, when clinically available, matched metastases. These cohorts should be annotated for anatomical subtype, etiology, growth pattern, molecular alteration, treatment history and recurrence pattern. Without such paired sampling, PMN claims in CCA will remain inferential.

Second, experimental models must incorporate organ-specific matrix and immunity. Patient-derived organoids, immune-organoid co-cultures, decellularized organ matrices, precision-cut tissue slices and humanized or syngeneic models can each address different aspects of metastasis. The decellularized lung and lymph node ECM organoid model demonstrates the value of recreating target-organ contexts, but immune components should be added to test whether ECM-induced epithelial states alter T cell killing, macrophage polarization or DC activation (73).

Third, mechanistic studies should prioritize intercellular nodes that connect multiple tissue compartments and determine whether perturbing one node rewires adjacent stromal and immune states. Such network-level experiments are more likely to identify durable therapeutic vulnerabilities than studies that evaluate isolated markers or cell populations independently.

Fourth, CCA subtype specificity must be addressed. Small-duct and large-duct intrahepatic CCA, perihilar CCA and distal CCA may differ in immune architecture, bile acid exposure, microbial signals, lymphatic anatomy and metastatic routes. A spatial metastasis framework should therefore avoid overgeneralization from intrahepatic CCA alone. Comparative studies across anatomical subtypes may reveal why some tumors recur locally, some spread to nodes and others colonize lung, peritoneum or bone.

Finally, clinical translation should be pragmatic. It may not be feasible to spatially profile every patient with multiple platforms, but a tiered approach is possible: routine histology and IHC to assess invasive-front architecture, multiplex panels for selected high-risk patients, blood-based EV or ctDNA assays for longitudinal monitoring and single-cell/spatial discovery studies embedded in trials. The goal is not technology for its own sake, but actionable classification of metastasis-enabling immune ecosystems. The proposed tiered workflow is consistent with the roadmap in **Figure 5** and the trial-design considerations in **Table 4**.

## 15 Conclusions

We propose a spatial immune-regulatory continuum as a hypothesis-generating framework for CCA metastasis rather than a fully validated causal model. Several highlighted mechanisms—including the POSTN+FAP+ CAF–SPP1+ TAM–MAIT cell triad, the bile acid–GPBAR1–CXCL10 axis and SAA1–CD36 nodal crosstalk—currently derive from single recent studies and require independent validation. The tumor core and invasive front create local conditions for dissemination through CAF-rich ECM, TAM and neutrophil programs, Treg-mediated suppression, DC defects and T cell exclusion. EVs, cytokines and lymphovascular routes may extend this influence to regional lymph nodes and distant organs. However, direct evidence that these signals establish organ-specific pre-metastatic niches before CCA-cell arrival remains lacking; current CCA data more directly support organ-specific adaptation during metastatic colonization. Established metastases then maintain immune escape through organ-specific cold ecosystems.

This model has two practical consequences. First, future CCA immunology studies should sample and analyze the invasive front, lymph nodes and metastases rather than treating the primary tumor as a homogeneous unit. Second, therapy should move beyond late-stage checkpoint rescue toward metastasis interception: reprogramming the invasive front, restoring antigen presentation, testing strategies that interrupt putative myeloid- and EV-mediated niche education and selecting combinations by spatial biomarkers. Such a strategy is ambitious and requires prospective validation, but it is consistent with current biological observations and the clinical need to prevent metastatic relapse.

## Glossary

CAF: cancer-associated fibroblast
CAR: chimeric antigen receptor
CAR-T: chimeric antigen receptor T-cell
CCA: cholangiocarcinoma
cDC1: type 1 conventional dendritic cell
ctDNA: circulating tumor DNA
DC: dendritic cell
ECM: extracellular matrix
EMT: epithelial-mesenchymal transition
EV: extracellular vesicle
FAP: fibroblast activation protein
FFPE: formalin-fixed, paraffin-embedded
FGFR: fibroblast growth factor receptor
FGFR2: fibroblast growth factor receptor 2
Flt3L: Fms-like tyrosine kinase 3 ligand
gMDSC: granulocytic myeloid-derived suppressor cell
HGF: hepatocyte growth factor
HLA: human leukocyte antigen
HSC: hepatic stellate cell
ICI: immune checkpoint inhibitor
iCAF: inflammatory cancer-associated fibroblast
iCCA: intrahepatic cholangiocarcinoma
IF: immunofluorescence
IHC: immunohistochemistry
IL: interleukin
LEC: lymphatic endothelial cell
LN: lymph node
LNM: lymph node metastasis
LOX: lysyl oxidase
MAIT cell: mucosal-associated invariant T cell
MDSC: myeloid-derived suppressor cell
MEOX1: mesenchyme homeobox 1
MFS: metastasis-free survival
mIF: multiplex immunofluorescence
MISEV: Minimal Information for Studies of Extracellular Vesicles
MRD: minimal residual disease
NK cell: natural killer cell
NET: neutrophil extracellular trap
PD-1: programmed cell death protein 1
PD-L1: programmed death-ligand 1
PDGF: platelet-derived growth factor
PDGFR: platelet-derived growth factor receptor
PMN: pre-metastatic niche
POSTN: periostin
RANKL: receptor activator of nuclear factor kappa-B ligand
scATAC-seq: single-cell assay for transposase-accessible chromatin sequencing
scRNA-seq: single-cell RNA sequencing
SAA1: serum amyloid A1
SPP1: secreted phosphoprotein 1
TAM: tumor-associated macrophage
TCR: T cell receptor
TGF-β: transforming growth factor beta
TLR: Toll-like receptor
TME: tumor microenvironment
Treg: regulatory T cell
VEGF: vascular endothelial growth factor
VEGFR: vascular endothelial growth factor receptor
